# DFT + *U* Study of Uranium Dioxide and Plutonium Dioxide with Occupation Matrix Control

**DOI:** 10.1021/acs.jpcc.2c03804

**Published:** 2022-07-01

**Authors:** Jia-Li Chen, Nikolas Kaltsoyannis

**Affiliations:** Department of Chemistry, School of Natural Sciences, University of Manchester, Oxford Road, Manchester M13 9PL, United Kingdom

## Abstract

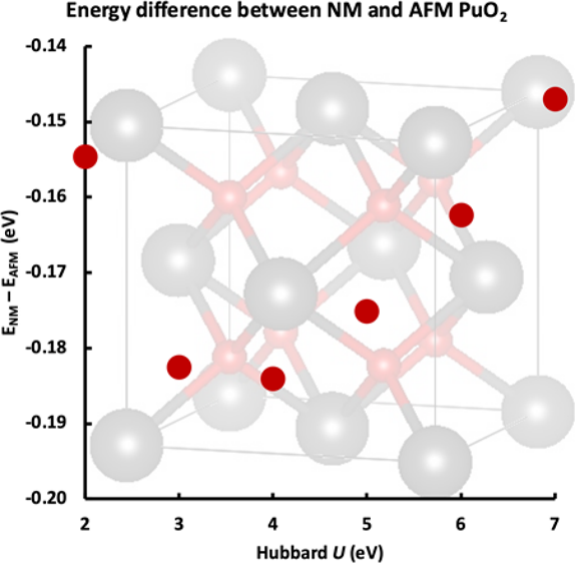

DFT + *U* with occupation matrix control (OMC) is applied to study computationally bulk UO_2_ and PuO_2_, the latter for the first time. Using the PBESol functional in conjunction with OMC locates AFM and NM ground states for UO_2_ and PuO_2_, respectively, in agreement with experimental findings. By simulating the lattice parameter, magnetic moment, band gap, and densities of states, *U* = 4.0 eV is recommended for AFM UO_2_, yielding data close to experiments for all considered properties. *U* = 4.5 and 4.0 eV are recommended for NM and AFM PuO_2_, respectively, though much larger *U* values (c. 10 eV) are required to yield the most recently reported PuO_2_ band gap. For both oxides, several excited states have similar properties to the ground state, reinforcing the need to employ OMC wherever possible.

## Introduction

In the actinide series of elements, many of the chemical and physical properties display a turning point at plutonium. This includes the change from the more covalent early actinides to the more ionic mid and later elements, and the extensive range of oxidation states exhibited by the early elements diminishes significantly after Pu.^1^ The actinide dioxides, which are the subject of this work, change from Mott–Hubbard insulators to charge transfer insulators at PuO_2_.^2−4^ UO_2_ and NpO_2_ have antiferromagnetic (AFM) ground states^5−7^ while a nonmagnetic (NM) ground state is found for PuO_2_.^8,9^ As PuO_2_ is a product of the recycling of spent UO_2_ nuclear fuel, detailed understanding of PuO_2_ is clearly essential not just at a fundamental level but also to inform its safe current and long-term storage.

Due to the high radioactivity of PuO_2_, experiments are very challenging, and hence theoretical simulations play a particularly valuable role in its study. Density functional theory (DFT) with a Hubbard *U* correction is widely used^4,10,11^ as it gives reasonable predictions at the lowest computational costs. However, the ability of the DFT + *U* approach to correctly identify the NM magnetic ground state of PuO_2_ remains an issue. The NM ground state has been established by various experiments over a wide temperature range (4–1000 K), including by inelastic neutron scattering and nuclear magnetic resonance.^8,9,12−14^ By contrast, previous DFT + *U* simulations have predicted an AFM ground state for PuO_2_;^11,15,16^ although this does not match with experiments, many of the other calculated properties of AFM PuO_2_ do agree quite well. There are also some other theoretical works that adopt the experimentally indicated NM state.^4,17,18^ The inconsistency between experiments and DFT + *U* simulation over the correct magnetic ground state of PuO_2_ requires further study.

The majority of actinide compounds are open-shell and frequently feature several unpaired electrons in the seven valence 5f orbitals. There are typically many different ways in which the actinide *f* orbitals may be populated, and use of the Hubbard *U* parameter in DFT calculations can lead to the location of excited states arising from those electronic configurations.^19,20^ It is unclear whether the previous computational reports of an AFM ground state for PuO_2_ arise from a fundamental inability of DFT + *U* to locate the correct ground state or if they have become trapped in higher energy states. To address this question, we here consider all possible filling patterns of the 4 electrons in the 7 5f orbitals of Pu(IV), using the occupation matrix control (OMC) approach.^21^ To the best of our knowledge, there is no such work so far. Wang and Konashi considered all possible occupation matrices (OMs) for AFM Pu (5f^5^) in PuO_2_,^22^ though not Pu (5f^4^) in which we are interested, and found that there are low-lying excited states. Dorado *et al.* considered all possible OMs for the 2 electrons in the 7 5f orbitals of AFM UO_2_,^23^ finding that the highest energy OM state is about 3.5 eV above the lowest one.

In this work, we first consider all possible OMs for AFM, FM, and NM UO_2_. Although previous DFT + *U* simulations agree with experiments over the AFM ground state for UO_2_,^24^ it is worth considering all possible OMs for FM and NM as well as AFM to be sure of the correct computed ground state for UO_2_, and UO_2_ provides a good test of the DFT + *U* with the OMC method as there are more data available on UO_2_. We then consider all possible OMs for AFM, FM, and NM PuO_2_, in order to establish the correct theoretical ground state. Through exploration of the effect of the choice of Hubbard *U* on a range of computed properties (lattice parameter, band gap, magnetic moment, and density of states), we also aim to provide recommendations as to the best values of *U* to employ in DFT + *U* + OMC studies of UO_2_ and PuO_2_.

## Computational Details

**Figure 1:**
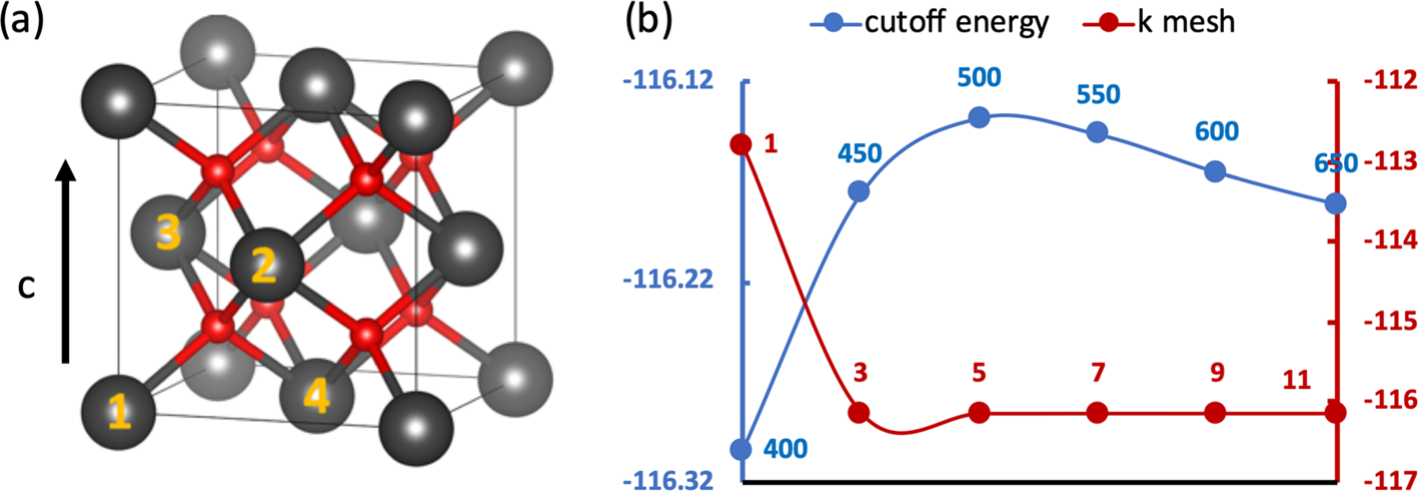
(a) UO_2_/PuO_2_ bulk, gray and red spheres represent U/Pu and O, respectively. (b) Energy against cutoff energy (*U* = 4.0 eV and *k* points = 5) and energy against *k* point (*U* = 4.0 eV and cutoff energy = 500 eV) for UO_2_ with a lattice parameter of 5.470 Å.

All calculations were performed using density functional theory (DFT), as implemented in the Vienna ab initio simulation package (VASP), version 5.4.1.^25−28^ The generalized gradient approximation functional of Perdew, Burke, and Ernzerhof, revised for solids (PBESol), was used,^29^ with a Hubbard *U* correction for the 5f electrons.^30^ A wide range of *U* values (0.0–7.0 eV) was considered to establish the most suitable values for the simulation of UO_2_ and PuO_2_ bulk (Figure 1a). Plane wave basis sets and projector augmented wave pseudopotentials were used to describe the ions.^31^ Plane wave cutoff energy and *k* mesh sizes were tested for UO_2_ bulk with the lattice parameter fixed at 5.470 Å (the experimental values are 5.470–5.473 Å);^32−34^Figure 1b shows that a plane wave cutoff of 500 eV and gamma-centered 5 × 5 × 5 Monkhorst–Pack grid for the Brillouin zone are sufficient.^35^ Therefore, a 650 eV (1.3 × 500 eV) cutoff energy (to weaken the influence of Pulay stress) and gamma-centered 5 × 5 × 5 Monkhorst–Pack grid were used for all calculations in this work. The iteration threshold for electronic and ionic convergence was set to 1 × 10^–5^ and 1 × 10^–2^ eV, respectively.

Antiferromagnetic (AFM), ferromagnetic (FM), and nonmagnetic (NM) states were considered with 1 *k* colinear ordering (along the *c* direction, Figure 1a) for both UO_2_ and PuO_2_. 1 *k* colinear ordering was chosen over 3 *k* non-colinear ordering because, although AnO_2_ exhibit non-collinear magnetic behavior^36^ in which the magnetic moments of the ions have contributions in more than one direction, 1 *k* ordering is much more computationally tractable than 2 *k* or 3 *k* ordering. Furthermore, most previous computational work also uses 1 *k* ordering, so our using it facilitates more direct comparison, and we here in part aim to find a theoretical approach that gives accurate simulation of UO_2_ and PuO_2_ at manageable computational cost. As the 1 *k* colinear ordering is used, we have only Type G magnetic arrangements (along the *c* direction). U/Pu labeled 1 and 2 in Figure 1a are set to spin up and 3 and 4 are set to spin down for all AFM calculations in this work.

Occupation matrix control (OMC), developed by Dorado *et al.*(23,37) and incorporated into VASP by Allen and Watson,^21^ was used to explore all possible OMs. Only the diagonal elements were set to non-zero values for initial OMs:
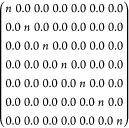
where *n* is either 0.0 or 1.0. U and Pu in their dioxide bulk have 2 and 4 5f electrons, respectively; these are unpaired in the FM and AFM states and paired in the NM state. Therefore, we studied *C*_7_^2^ = 21 OMs for FM and AFM UO_2_, *C*_7_^1^ = 7 OMs for NM UO_2_, *C*_7_^4^ = 35 OMs for FM and AFM PuO_2_, and *C*_7_^2^ = 21 OMs for NM PuO_2_; all OMs are listed in the Supporting Information (Tables S1 and S2). Although some of the electronic configurations defined by the OMs are degenerate, we decided to follow the approach of Dorado *et al.*, who used DFT + *U* + OMC to investigate bulk UO_2_^23^ and study all the OMs as the imposition of *U* can decrease the degeneracy of the f orbitals. The initially imposed OMs remain unchanged during the self-consistent field calculations.

All of the data presented in the main text were obtained with the PBESol functional. We also performed all the calculations using the PBE functional and found that PBESol predicts better lattice parameters. The PBE data are collected in Figures S5–S9 and Table S3 in the Supporting Information.

## Results and Discussion

### Uranium Dioxide (UO_2_)

**Figure 2:**
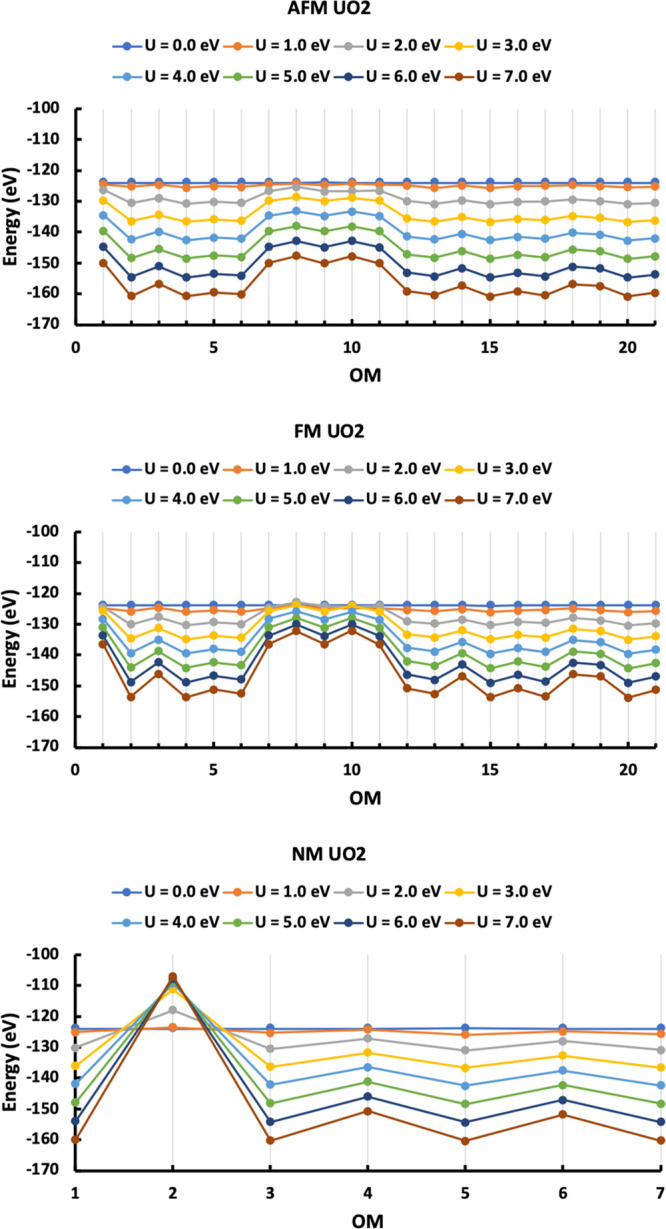
energies of all solutions of AFM, FM and NM UO_2_ as a function of *U* from 0.0 eV to 7.0 eV. OMs are listed in Table S1.

We now explore the energies of the AFM, FM, and NM states of UO_2_ with the OMs as described in the Computational Details. As the Hubbard *U* has an influence on the localization and energy of the *f* orbitals, a wide range of *U* values is considered. The pure PBEsol method (*U* = 0.0 eV) predicts the same energy for all solutions of AFM, FM and NM UO_2_ (Figure 2). Furthermore, checking the magnetic moment on each U atom indicates that all AFM and NM states optimize to FM states. Introduction of a non-zero *U* value breaks the degeneracy of the *f* orbitals obtained from the pure DFT simulation, the data for *U* = 1.0 to 7.0 eV in Figure 2 are no longer linear, and stable AFM and NM solutions are found.

The effect of the Hubbard *U* becomes increasingly clear with increasing *U* value. Energy differences between different solutions are amplified at high *U*, as wider energy ranges of solutions are found. Small *U* values (<2 eV) are not enough to overcome the drawback of pure DFT as some AFM and NM states still optimize to FM states, but larger *U* values (≥ 2 eV) predict the same OM for the most stable solution. For AFM and FM, the OM has two unpaired electrons occupying the f_1_ and f_3_ orbitals (the 20th OM listed in Table S1), while for the NM state, the OM has pair of electrons in the f_1_ orbital (the 5th OM listed in Table S1). In the following discussion, we focus on the most stable AFM, FM, and NM OM solutions, and refer to them as the AFM, FM, and NM states for simplicity.

**Figure 3:**
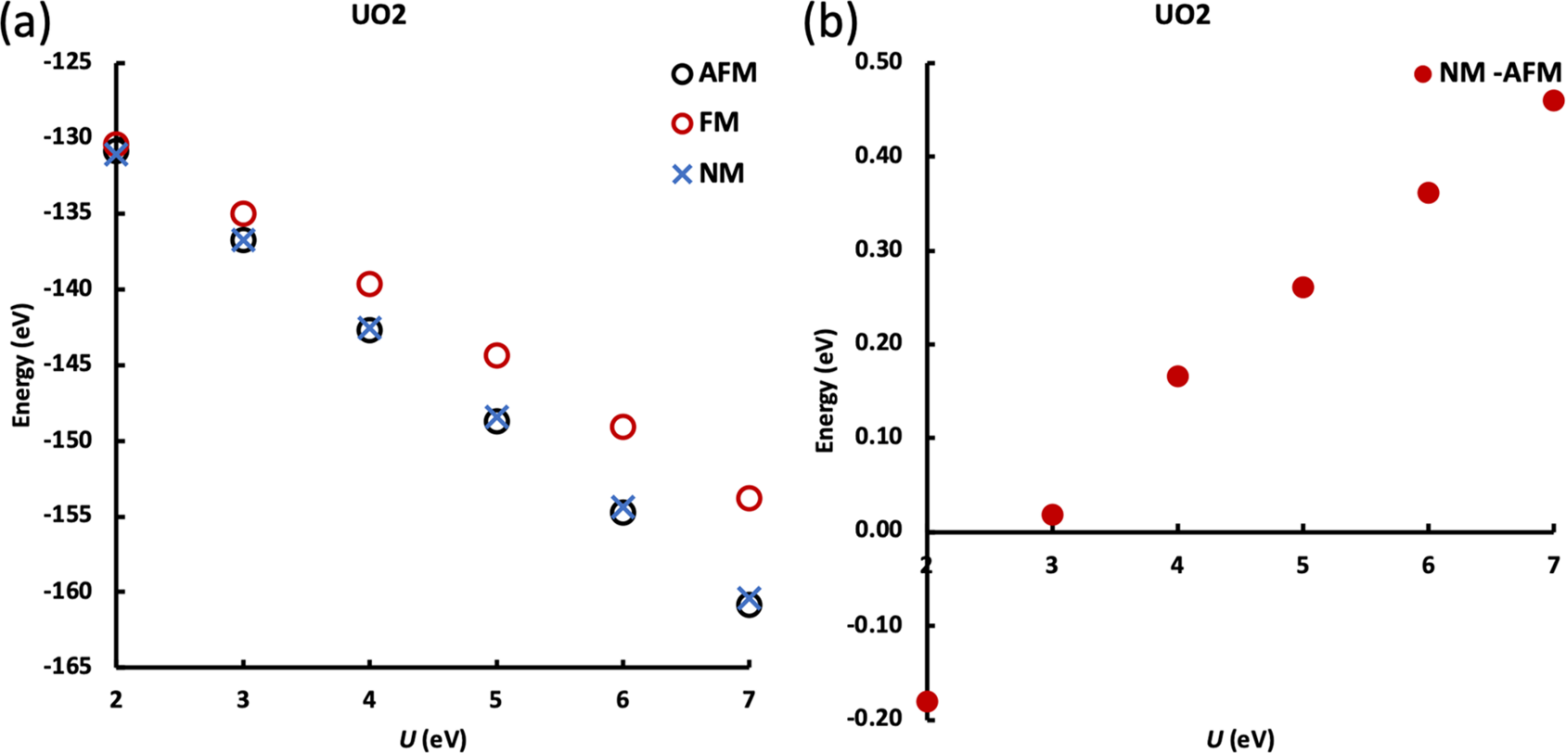
(a) Energies of AFM, FM, and NM UO_2_ as a function of *U*. (b) Energy difference between AFM and NM UO_2_ (*E*_NM_ – *E*_AFM_) as a function of *U*.

As noted above, pure DFT and low *U* values (<2.0 eV) predict an FM ground state, regardless of the initially chosen magnetic state; the energies obtained with *U* ≥ 2.0 eV are compared for AFM, FM, and NM states in Figure 3. As shown in Figure 3b, an NM ground state is found for UO_2_ with 2.0 ≤ *U* < 3.0 eV; when *U* ≥ 3.0 eV, the AFM ground state appears. Previous experiments have confirmed that the ground state of UO_2_ is AFM, so the chosen *U* value should be not smaller than 3.0 eV to give reasonable prediction. As the ground state is predicted differently with low and high *U* values, we suggest caution when employing the *U*-ramping method, in which *U* is scanned from zero to the desired value in small steps, while reading the previous step’s result.

**Figure 4:**
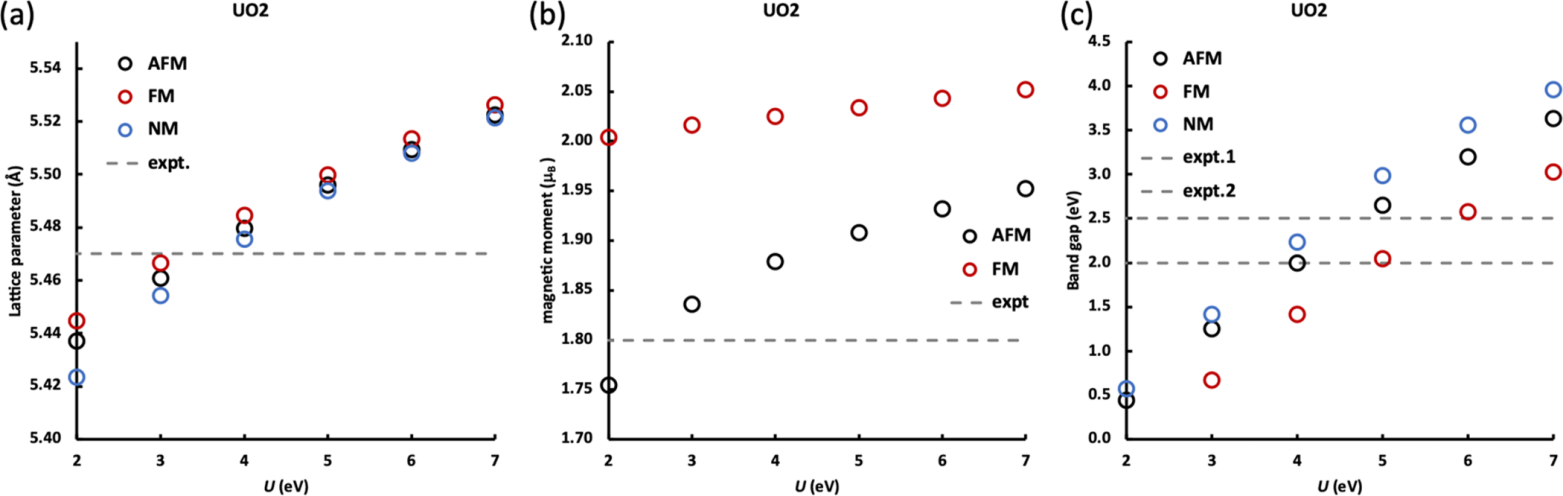
(a) Lattice parameter of AFM, FM, and NM UO_2_, with experimental values from refs (32, 34). (b) Magnetic moment of U in AFM and FM UO_2_, with experimental value from ref (38). (c) Band gap of AFM, FM, and NM UO_2_, with experimental values from refs (39, 40).

We did not find any distortion of cubic UO_2_ bulk, i.e., 1 *k* AFM UO_2_ keeps the *Fm*-3*m* crystal symmetry. The lattice parameter gradually increases with the *U* value, and 3 eV ≤ *U* ≤ 4 eV yields lattice parameters close to the experimental values (Figure 4a). The magnetic moment on the U ions also increases with increasing *U* (Figure 4b); imposing *U* on the f orbitals leads to more localized f electrons. AFM and FM UO_2_ simulations slightly overestimate the magnetic moment on U ions, but the differences are small, and AFM UO_2_ is predicted to have a magnetic moment closer to the experimental value.^38^ The band gap of UO_2_ increases with *U* in a similar way for the AFM, FM, and NM states. Across the range of *U* studied, NM UO_2_ has the largest band gap and FM UO_2_ has the smallest band gap, with the band gap of AFM UO_2_ being slightly lower than for NM. Figure 4c suggests that 4.0 eV ≤ *U* ≤ 5.0 eV gives good agreement with experiments for the band gap of AFM UO_2_.^39,40^

**Figure 5:**
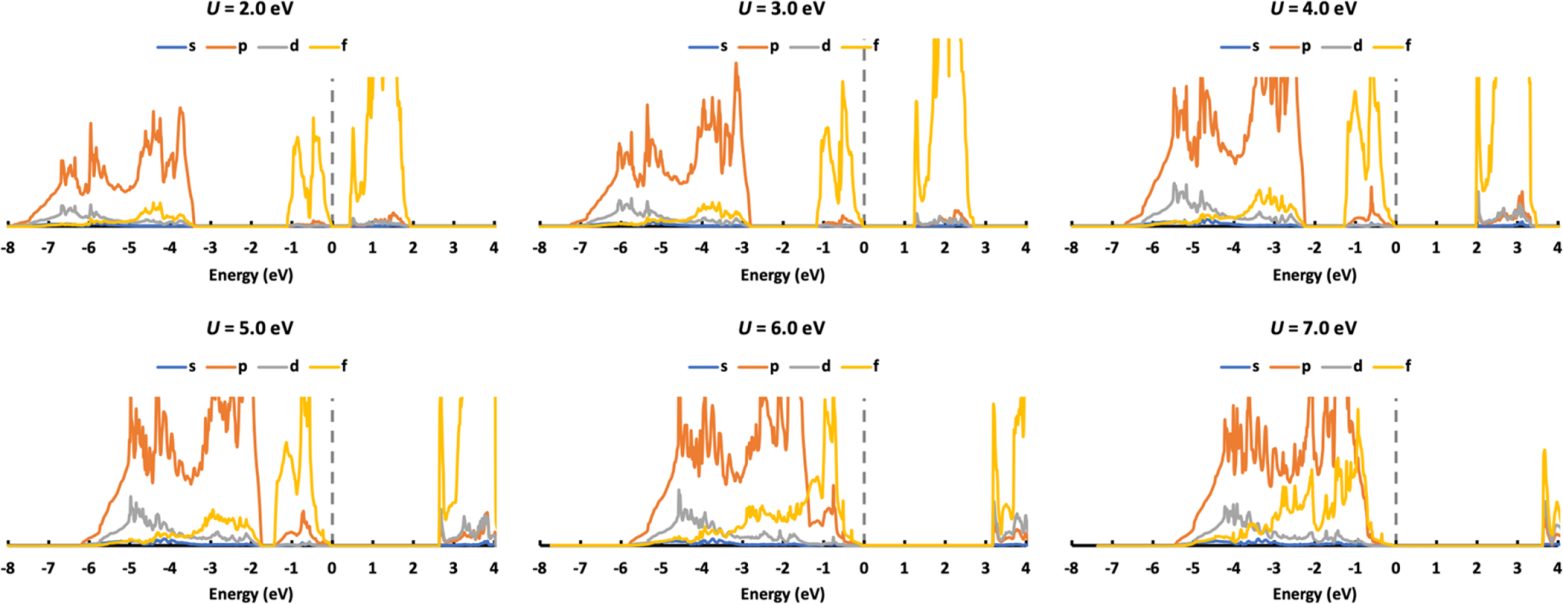
DOS of AFM UO_2_ calculated with *U* values ranging from 2.0 to 7.0 eV.

As well as the band gap, the density of states (DOS) is an important electronic property against which to evaluate the simulation. As AFM is the experimentally and theoretically reported ground state, only the DOS of AFM UO_2_ is discussed here. We plot the DOS for AFM UO_2_ with *U* values ranging from 2.0 to 7.0 eV in steps of 1.0 eV (Figure 5). We are mainly interested in three bands in the DOS: the valence band, the conduction band, and the second band below the Fermi level. Increasing the Hubbard *U* localizes and stabilizes the f electrons. The valence band, which is mainly U 5f, moves downward in the DOS. This increases the band gap, and the gap between the valence band and the second band (which is mainly of O 2p character) reduces, such that the two bands become mixed at high *U* (6.0 and 7.0 eV). As the Fermi level is fixed at 0.0 eV, the conduction band and the second band under the Fermi level move upward with increasing *U* value.

X-ray absorption data indicate that the valence band is mainly U 5f,^41^ so we can exclude *U* ≥ 6.0 eV. Previous experiments also show that the second peak under the Fermi level consists of O 2*p* states at around −4.0 eV,^41,42^ so we can also exclude *U* = 5.0 eV. Though *U* = 2.0 eV predicts a reasonable position for the second peak under the Fermi level, the band gap is much smaller than the experimental value and it predicts an NM ground state. Therefore, 2.0 eV < *U* < 5.0 eV is the preferred range to obtain reasonable DOS for UO_2_ bulk simulation.

Overall, we find that 3.0 eV ≤ *U* ≤ 5.0 eV gives the best balance of agreement with experimental data over a range of properties, with the best value being 4.0 eV. Some properties of AFM UO_2_ calculated with the PBESol + *U* (*U* = 4.0 eV) + OMC method are summarized and compared with experimental values in Table 1.

**Table 1:** Bulk Properties of AFM UO_2_, NM PuO_2_, and AFM PuO_2_ from Experiments (Experimental Values for UO_2_ from refs (32, 34, 42, 43), Experimental Values for PuO_2_ from refs (34, 48))^a^

	AFM UO_2_		NM PuO_2_		AFM PuO_2_
	expt.	cal.	expt.	cal.	cal.
lattice parameter (Å)	5.470–5.473	5.479	5.393–5.398	5.394	5.390
magnetic moment (μ_B_)	1.74	1.88	0	0	3.72
band gap (eV)	2.0–2.5	2.0	1.8–4.1	1.56	0.9

a Data obtained with PBESol + *U* + OMC (*U* = 4.0 eV for AFM UO_2_ and AFM PuO_2_, 4.5 eV for NM PuO_2_). The equivalent PBE data can be found in Table S3.

### Plutonium Dioxide

We now explore AFM, FM, and NM PuO_2_ calculated with the OMs described in the Computational Details, with the PBESol + *U* (*U* = 0.0–7.0 eV) method. The energies of these states are summarized in Figure S1. As for UO_2_, *U* values ≥2.0 eV predict the same solution as the most stable: for AFM and FM, the most stable solution has the four unpaired electrons occupying the f_–3_, f_–1_, f_1_, and f_3_ orbitals (the 31st OM in Table S2), while for the NM states, the most stable solution has two pairs of electrons in the f_1_ and f_3_ orbitals (the 20th OM in Table S2). In the following discussion, we focus on the most stable AFM, FM, and NM solutions and refer to them as the AFM, FM, and NM states for simplicity.

**Figure 6:**
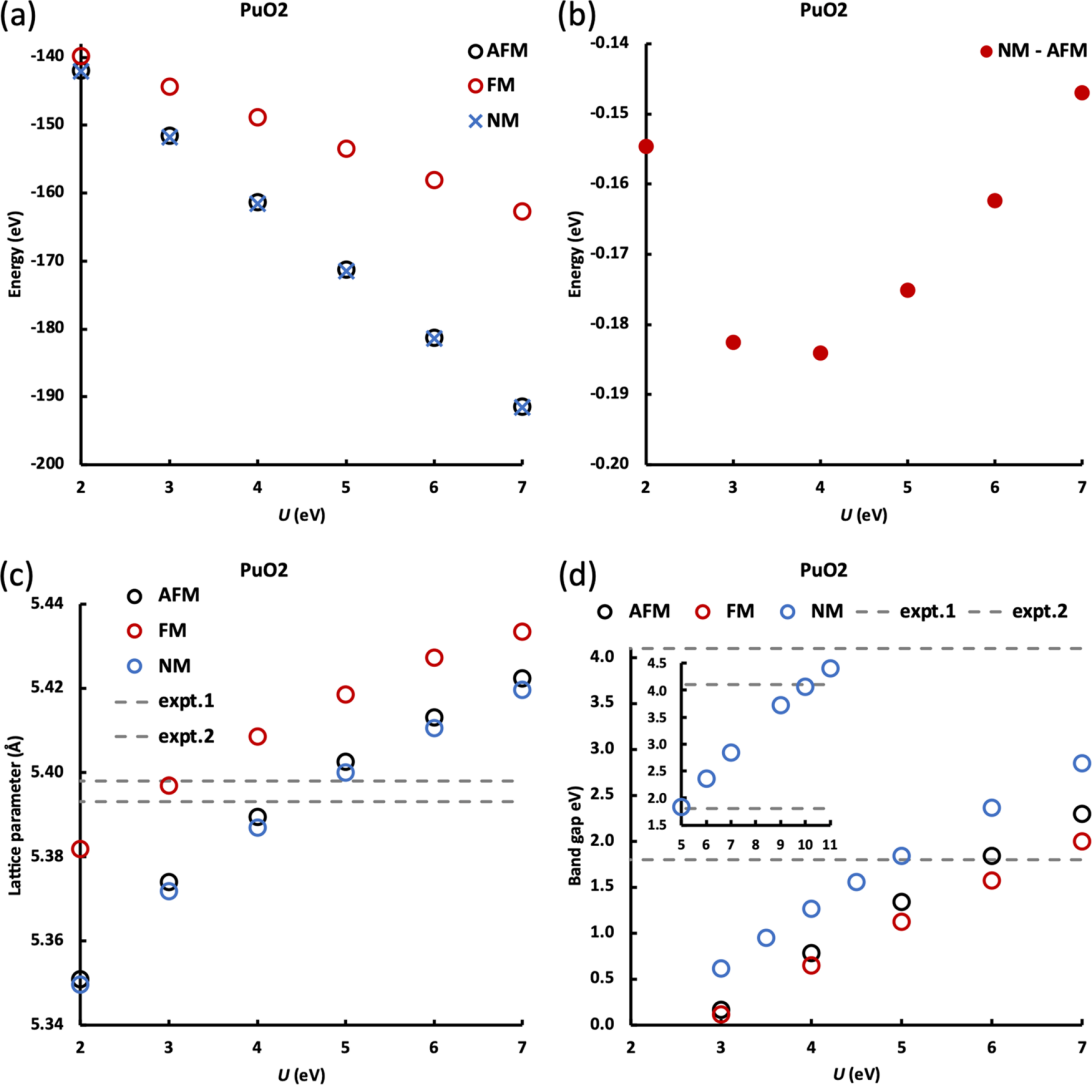
As a function of *U*, the (a) energies of AFM, FM, and NM PuO_2_, (b) energy difference between AFM and NM PuO_2_ (*E*_NM_ – *E*_AFM_), (c) lattice parameter of AFM, FM, and NM PuO_2_, experimental values from refs (34, 43, 46), and (d) band gap of AFM, FM, and NM PuO_2_, experimental values from refs (38, 47). Inset to panel (d) shows the band gap of NM PuO_2_ calculated with *U* = 5–11 eV.

Properties of the AFM, FM, and NM states are compared in Figure 6. When *U* ≥ 2.0 eV, an NM ground state is found, in agreement with previous experiments in the temperature range 4–1000 K.^8,14^ However, the energy difference between AFM and NM is small (Figure 6b), increasing from *U* = 2.0 to 4.0 eV and then decreasing again, with the largest difference of −0.18 eV. Previous DFT + *U* simulations have found an AFM ground state for PuO_2_, although given the small energy differences with NM states, it may be that DFT + *U* simulation of PuO_2_ without OMC can become trapped in an AFM state.

Similar to UO_2_, PuO_2_ bulk remains face-centered cubic after optimization and the optimized lattice parameter increases with the *U* value (Figure 6c), with 4.0 eV ≤ *U* ≤ 5.0 eV giving values close to experiments for AFM and NM.^34,43−46^ These states have similar lattice parameters for a given *U*, while a much larger lattice parameter is predicted for FM PuO_2_ with the same *U* value. This is by contrast to UO_2_, where although the largest lattice parameter was also obtained for the FM state, it is close to the AFM and NM lattice parameters for a given *U* value (Figure 4a). This may well be reminiscent of previous work on the paramagnetic to ferromagnetic transition of La(Fe_*x*_Si_1–*x*_)_13_,^47^ where a larger volume change is observed for *x* = 0.88 (where Fe has a larger magnetic moment) than for *x* = 0.86 (where Fe has a smaller magnetic moment); as Pu^4+^ has a larger magnetic moment than U^4+^, the difference between the lattice parameter of FM and AFM/NM for PuO_2_ is larger than for UO_2_.

PuO_2_ is found to be nonmagnetic by experiment, and hence the magnetic moment of Pu should be zero, and experiments indeed find only a very small nuclear magnetic moment for Pu^4+^ (about 0.15 μ_N_),^14^ which may arise from coupling between the singlet Γ_1_ ground state and (an) excited state(s). The excited state could be solely the triplet Γ_4_ state, at an energy of about 0.120 eV, or two or more states in the energy region 0.110–0.140 eV, but spectral resolution is insufficient to be certain.^9^ We here find that AFM PuO_2_ is higher in energy than NM PuO_2_ by less than 0.18 eV, so ground-state PuO_2_ could have a small contribution from the AFM state; high-resolution neutron spectroscopy would be helpful here. The Pu magnetic moments of AFM and FM PuO_2_ against the *U* value are given in Figure S2; due to more localized f states with increasing *U* value, the magnetic moment of Pu increases with *U*.

Pure PBESol and PBESol + *U* with a small *U* value (< 3.0 eV) predict PuO_2_ to be metallic; with increasing *U*, PuO_2_ becomes a semiconductor with a band gap that increases with *U*. As with UO_2_, NM PuO_2_ has the largest band gap, and FM has the smallest. 5.0 ≤ *U* ≤10.0 eV are needed to give band gaps in the range of the experimental data (1.8 eV–4.1 eV),^46,48,49^ see inset to Figure 6d.

**Figure 7:**
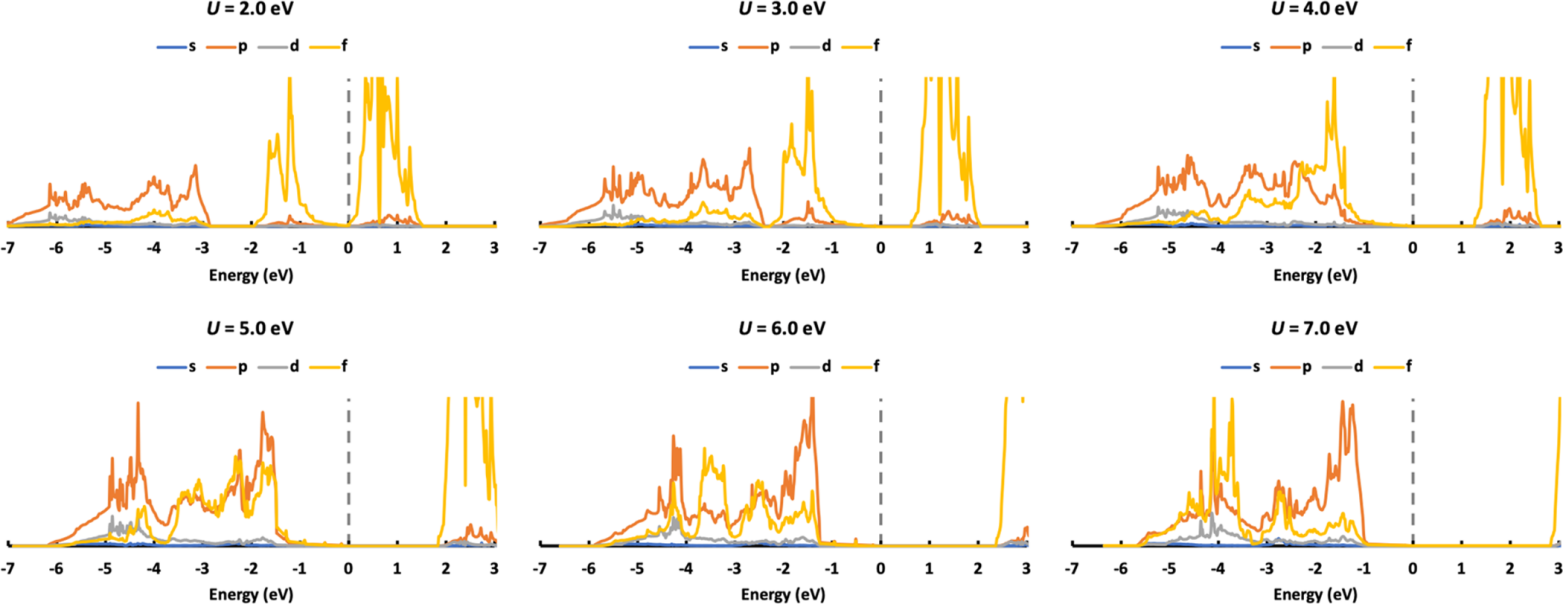
DOS of NM PuO_2_ calculated with *U* values ranging from 2.0 to 7.0 eV.

We now examine the DOS of the NM PuO_2_ ground state (Figure 7). As with UO_2_, we are interested in the conduction and valence bands as well as the second band under the Fermi level. Different from UO_2_, there is a mix of p and f states in both the valence band and the second band under the Fermi level, although similar to the DOS of UO_2_, increasing *U* leads to the downward movement of the valence band while upward movement of the conduction band and second band under the Fermi level is observed in Figure 7. Downward movement of the valence band results in a larger band gap and more complicated interaction between the valence band and the second band under the Fermi level. When *U* < 4.0 eV, the valence band moves downward and approaches the second band, but there is a clear boundary to the position of the valence band (f state-dominated and at around −2 to 0 eV) and the second band (p state-dominated and at around −7 to −2 eV). When U ≥ 4.0 eV, the origin of the second band and valence band are merged; a new valence band (at around −4 to 0 eV) and a new second band under the Fermi level (p state-dominated and at around −7 to −4 eV, though it is connected with the valence band, we name it is as the second band under the Fermi level to distinguish it from the abovementioned new valence band) are observed. The new valence band has a similar contribution from the p and f states. When *U* ≥ 5.0 eV, O p states gradually dominate the valence.

The valence band of PuO_2_ is o mixed f and p character, with a higher contribution of the former,^50−52^ so *U* ≥ 5.0 eV values are not good choices as they predict a higher (or equal) contribution of p states than f states for the valence band. Previous X-ray photoelectron spectra have shown that the valence band is split into a mainly Pu 5f-contributed state (at higher energy) and a mainly O 2p-contributed state (at lower energy),^50^ which is also supported by previous theoretical simulations. Previous UPS studies showed that the contribution of Pu 5f states is centered at around 2 eV below the Fermi level, in agreement with XPS data.^51^ XPS also shows that the mainly O 2p band, which covers a wide range below the Fermi level, extends to −10 eV,^50^ while it ends at around −8 eV from UPS data.^51^

**Figure 8:**
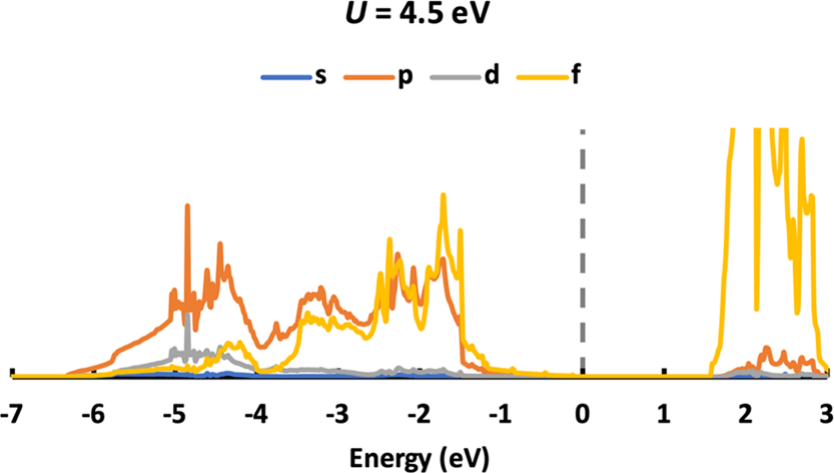
DOS of NM PuO_2_ calculated with PBESol + *U* (4.5 eV) + OMC.

The center of the Pu 5f states moves downward with increasing *U*; for NM PuO_2_ simulated with *U* = 2.0 eV, the 5f states are centered at around −1.5 eV, at around −2.0 eV with *U* = 4.0 eV, matching the experiment well, and at around −2.5 eV with *U* = 5.0 eV, which is also reasonable. The O 2p states end at around −7 eV for NM PuO_2_ with *U* = 2.0 eV, which is the closest value to experimental data (about −8 eV), as the second band moves upward with increasing *U*, ending at around −6 eV for NM PuO_2_ with *U* = 5.0 eV. In general, 4.0 eV ≤ *U* < 5.0 eV gives reasonable prediction for the position of the Pu 5f states in the valence band (Figure 7). We have also studied NM PuO_2_ bulk with *U* = 4.5 eV; the DOS is given in Figure 8, which meets the experimentally reported features well, such as the composition and center of the valence band. Previous works also suggest that there is a peak of the O p state character on the left shoulder of the valence band, a feature which can be seen in our DOS for NM PuO_2_ simulated with *U* = 4.0 and 4.5 eV. Overall, 4.0 eV ≤ *U* ≤ 4.5 eV is good for NM PuO_2_ bulk simulation with *U* = 4.5 eV being the best, as this value predicts a larger band gap than *U* = 4.0 eV.

**Figure 9:**
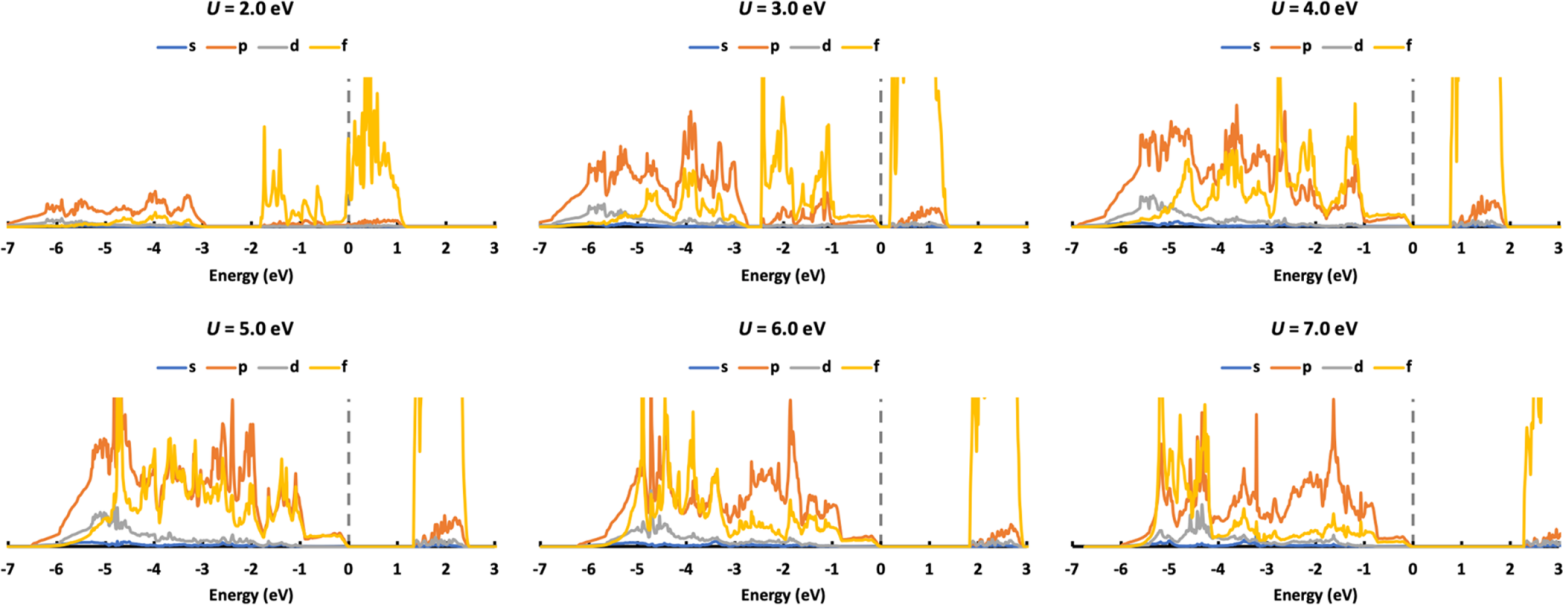
DOS of AFM PuO_2_ calculated with *U* values ranging from 2.0 to 7.0 eV.

We also studied the DOS of AFM PuO_2_ as it is the most studied state of PuO_2_ and may have a contribution to the ground state. The DOS of AFM PuO_2_ (Figure 9) is similar to those of NM PuO_2_, with some minor differences. The DOS of AFM PuO_2_ shown here is also similar to previous theoretical simulations. Therefore, although most previous DFT + *U* works without OMC study the AFM state of PuO_2_, they still obtain results similar to experiments, i.e., AFM PuO_2_ is a good approximation to NM PuO_2_ in the simulation of certain properties. To obtain good DOS for AFM PuO_2_, the *U* value should be smaller than 5 eV as *U* ≥ 5.0 eV predicts an O 2p state-dominated valence band. The DOS of PuO_2_ calculated with *U* = 4.5 eV is given in Figure S3 and predicts almost the same contribution of p and f states to the valence band, so *U* ≤ 4.5 eV is needed to predict an f state-dominated valence band, while *U* < 3.0 eV predicts AFM PuO_2_ as metallic. Overall, for AFM PuO_2_, 4.0 eV ≤ *U* ≤ 4.5 eV with *U* = 4.0 eV is the best as it predicts more reasonable DOS.

In summary, first, to obtain an NM ground state for PuO_2_, the chosen *U* value must be larger than 2 eV; second, to obtain reasonable lattice parameters, 4.0 eV ≤ *U* ≤ 5.0 eV is suggested; third, 5.0 eV ≤ *U* ≤ 10.0 eV is needed to reproduce the range of experimentally reported band gaps; fourth, to give reasonable DOS, 4.0 eV ≤ *U* ≤ 5.0 eV is suggested. Hence, no single *U* value can simultaneously reproduce the band gap and the other three pieces of experimental data. *U* = 10.0 predicts a band gap of 4.10 eV, which is the latest experimental datum, but it also predicts totally wrong DOS (Figure S4). Overall, therefore, we suggest 4.0 eV ≤ *U* ≤ 5.0 eV with 4.5 and 4.0 eV being the best values for the simulation of NM and AFM PuO_2_ bulk, respectively. Some properties of NM and AFM PuO_2_ calculated with PBESol + *U* (4.5/4.0 eV) + OMC are listed in Table 1.

### Dependence of Energy, Lattice Parameter, and Band Gap on the Occupation Matrix

With the ground state OMs and the effect of the Hubbard *U* on the properties of those ground states in hand for both UO_2_ and PuO_2_, we here provide an insight into how the solutions calculated from different initial OMs affect the energy, lattice parameter, and band gap. Table 2 presents these data for the solutions arising from the different initial OMs of AFM UO_2_ and NM PuO_2_, using *U* values of 4.0 and 4.5 eV, respectively.

**Table 2:**
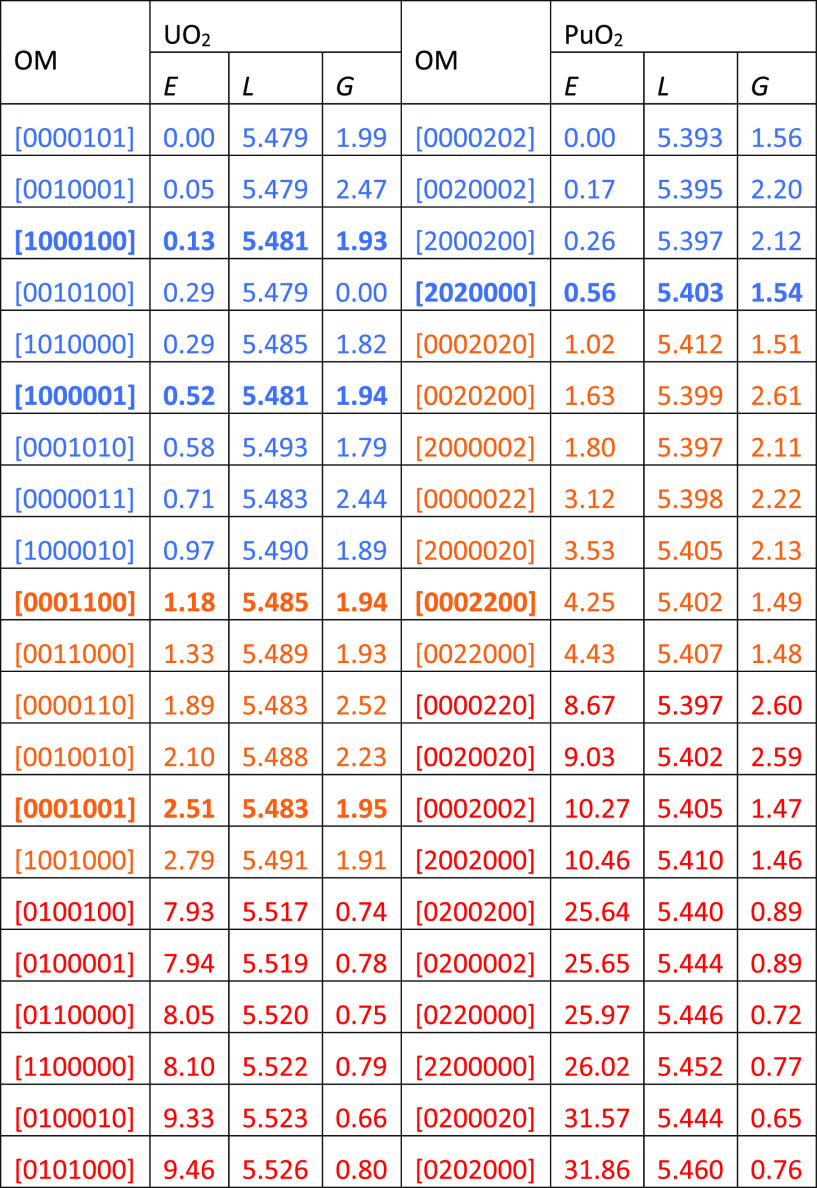
Occupation Matrix (OM, Details in Tables S1 and S2), Relative Energy (*E*, eV), Lattice Parameter (*L*, Å), and Band Gap (*G*, eV) of Each Solution of AFM UO_2_ and NM PuO_2_ Calculated with PBESol + *U* (4.0 eV and 4.5 eV, Respectively)^a^

a Low-, medium- and high-energy solutions are in given blue, orange, and red, respectively.

We find the same OM for ground-state AFM UO_2_ as does previous work,^23^ and the energy range spanned by the solutions is about 9 eV. This is significantly larger than in the previous study, where the highest energy solution is only 3.45 eV higher than the ground state, although we note that previous workers report some non-converged states, which have the same OMs as the high-energy solutions (Table 2, in red) we find here. These high-energy solutions have a slightly larger lattice parameter (∼5.53 Å) than the ground state (5.479 Å) and much smaller band gaps (∼1.0 eV *vs* 1.99 eV). We expect that, due to their high energy, optimizations without initial OMC have a good chance of avoiding these solutions.

There are 6 medium-energy solutions of AFM UO_2_ with relative energies of c. 1 to 3 eV (Table 2, in orange) and a further 8 low-energy solutions with relative energies of <1 eV (Table 2, in blue). Given these small relative energies, optimization of AFM UO_2_ could well become trapped in any of these low- to medium-energy solutions. Some medium- and low-energy solutions predict significantly higher band gaps than the ground state, *e.g.*, the [0010001] solution has a band gap of 2.47 eV. The remaining two medium energy solutions ([0001100] and [0001001]) and low-energy solutions ([1000100] and [1000001]) (Table 2, in bold) have lattice parameters and band gaps close to those of the ground state; indeed, it is hard to distinguish them from the ground state without OMC.

For NM PuO_2_, there are 10 (Table 2, in red), 7 (Table 2, in orange), and 4 (Table 2, in blue) high-, medium-, and low-energy solutions, respectively. The high-energy solutions have relative energies of c. 7–28 eV, i.e., they are very unstable, while the medium- and low-energy solutions have relative energies of c. 1–4 eV and < 1 eV, respectively. Most of the medium- and low-energy solutions of NM PuO_2_ have significantly larger or smaller band gaps than the ground state; there is only one medium-energy solution ([0002200]) and one low-energy solution ([2020000]) with similar lattice parameters and band gaps to the ground state.

Overall, for both oxides, there are some solutions with similar energies and lattice parameters to the ground state but with very different band gaps. However, other states have similar energies, lattice parameters, and band gaps to the ground state, and hence it is very hard to distinguish them from the ground state without OMC.

## Conclusions

In this work, we have studied bulk UO_2_ and PuO_2_ with PBESol + *U* (0–7 eV) + OMC. By calculating the energies of all possible solutions with different initially imposed OMs of 1 *k* AFM, FM and NM UO_2_ and PuO_2_, PBESol + *U* + OMC simulation predicts AFM and NM ground states for UO_2_ and PuO_2_, respectively. Our UO_2_ ground state is in agreement with previous experimental results and theoretical simulations. For PuO_2_, we show for the first time that PBESol + *U* + OMC correctly reproduces the experimentally reported NM ground state. We have also considered a wide range of *U* in order to find the best value for theoretical simulation. The lattice parameter, magnetic moment, band gap, and density of states have been simulated. *U* = 4.0 eV is recommended for AFM UO_2_, as this gives data close to experiments for all considered properties. For NM and AFM PuO_2_, we recommend *U* = 4.5 and 4.0 eV, respectively, though note that extremely large *U* values (c. 10 eV) are required to yield the most recently reported PuO_2_ band gap. Exploration of the energies, lattice parameters, and band gaps of AFM UO_2_ and NM PuO_2_ calculated with PBESol + *U* (4.0 and 4.5 eV, respectively) + OMC reveals that several excited states have similar properties to the ground state and hence it is very hard to distinguish them from the ground state in the absence of OMC.

## Supporting Information Available

Occupation matrices of U and Pu 5f orbitals for AFM, FM, and NM UO_2_ and PuO_2_; energies of AFM, FM, and NM PuO_2_; magnetic moment of Pu in AFM and FM PuO_2_; DOS of AFM PuO_2_ calculated with PBESol + *U* (4.5 eV) + OMC; DOS of NM PuO_2_ calculated with PBESol + *U* (10.0 eV) + OMC; PBE + *U* + OMC results for UO_2_ and PuO_2_ (PDF)

## Notes

The data underlying this study are openly available at https://data.mendeley.com/datasets/zrcv3yw9sn/1.
