# Supplementary material for: DFT + U Study of Uranium Dioxide and Plutonium Dioxide with Occupation Matrix Control
Source: J Phys Chem C Nanomater Interfaces. 2022 Jul 1;126(27):11426–35. doi: 10.1021/acs.jpcc.2c03804 (PMC9289946; doi:10.1021/acs.jpcc.2c03804)
Supplement: Supplementary file 1 — jp2c03804_si_001.pdf [file jp2c03804_si_001.pdf]

DFT + *U* Study of Uranium Dioxide and Plutonium Dioxide with Occupation Matrix Control

Jia-Li Chen and Nikolas Kaltsoyannis*

Department of Chemistry, School of Natural Sciences, University of Manchester, Oxford Road, Manchester M13 9PL, United Kingdom

ORCID
Jia-Li Chen: 0000-0003-2964-0127
Nikolas Kaltsoyannis: 0000-0003-0293-5742

* Email: nikolas.kaltsoyannis@manchester.ac.uk

Table S1: 5*f* orbital occupation matrices for AFM, FM and NM UO_2.

NO.	OM	AFM/FM							OM	NM						
		f_{-3}	f_{-2}	f_{-1}	f_0	f_1	f_2	f_3		f_{-3}	f_{-2}	f_{-1}	f_0	f_1	f_2	f_3
1	[1100000]	1	1	0	0	0	0	0	[2000000]	2	0	0	0	0	0	0
2	[1010000]	1	0	1	0	0	0	0	[0200000]	0	2	0	0	0	0	0
3	[1001000]	1	0	0	1	0	0	0	[0020000]	0	0	2	0	0	0	0
4	[1000100]	1	0	0	0	1	0	0	[0002000]	0	0	0	2	0	0	0
5	[1000010]	1	0	0	0	0	1	0	[0000200]	0	0	0	0	2	0	0
6	[1000001]	1	0	0	0	0	0	1	[0000020]	0	0	0	0	0	2	0
7	[0110000]	0	1	1	0	0	0	0	[0000002]	0	0	0	0	0	0	2
8	[0101000]	0	1	0	1	0	0	0								
9	[0100100]	0	1	0	0	1	0	0								
10	[0100010]	0	1	0	0	0	1	0								
11	[0100001]	0	1	0	0	0	0	1								
12	[0011000]	0	0	1	1	0	0	0								
13	[0010100]	0	0	1	0	1	0	0								
14	[0010010]	0	0	1	0	0	1	0								
15	[0010001]	0	0	1	0	0	0	1								
16	[0001100]	0	0	0	1	1	0	0								
17	[0001010]	0	0	0	1	0	1	0								
18	[0001001]	0	0	0	1	0	0	1								
19	[0000110]	0	0	0	0	1	1	0								
20	[0000101]	0	0	0	0	1	0	1								
21	[0000011]	0	0	0	0	0	1	1								

Table S2: 5*f* orbital occupation matrices for AFM, FM and NM PuO_2.

NO.	AFM/FM								NM							
	OM	f_{-3}	f_{-2}	f_{-1}	f_0	f_1	f_2	f_3	OM	f_{-3}	f_{-2}	f_{-1}	f_0	f_1	f_2	f_3
1	[1110100]	1	1	1	0	1	0	0	[2200000]	2	2	0	0	0	0	0
2	[1110010]	1	1	1	0	0	1	0	[2020000]	2	0	2	0	0	0	0
3	[1110001]	1	1	1	0	0	0	1	[2002000]	2	0	0	2	0	0	0
4	[0111010]	0	1	1	1	0	1	0	[2000200]	2	0	0	0	2	0	0
5	[0111001]	0	1	1	1	0	0	1	[2000020]	2	0	0	0	0	2	0
6	[0011101]	0	0	1	1	1	0	1	[2000002]	2	0	0	0	0	0	2
7	[1011100]	1	0	1	1	1	0	0	[0220000]	0	2	2	0	0	0	0
8	[1001110]	1	0	0	1	1	1	0	[0202000]	0	2	0	2	0	0	0
9	[0101110]	0	1	0	1	1	1	0	[0200200]	0	2	0	0	2	0	0
10	[1000111]	1	0	0	0	1	1	1	[0200020]	0	2	0	0	0	2	0
11	[0100111]	0	1	0	0	1	1	1	[0200002]	0	2	0	0	0	0	2
12	[0010111]	0	0	1	0	1	1	1	[0022000]	0	0	2	2	0	0	0
13	[1101100]	1	1	0	1	1	0	0	[0020200]	0	0	2	0	2	0	0
14	[1100110]	1	1	0	0	1	1	0	[0020020]	0	0	2	0	0	2	0
15	[1100011]	1	1	0	0	0	1	1	[0020002]	0	0	2	0	0	0	2
16	[0110110]	0	1	1	0	1	1	0	[0002200]	0	0	0	2	2	0	0
17	[0110011]	0	1	1	0	0	1	1	[0002020]	0	0	0	2	0	2	0
18	[0011011]	0	0	1	1	0	1	1	[0002002]	0	0	0	2	0	0	2
19	[1101010]	1	1	0	1	0	1	0	[0000220]	0	0	0	0	2	2	0
20	[1101001]	1	1	0	1	0	0	1	[0000202]	0	0	0	0	2	0	2
21	[1100101]	1	1	0	0	1	0	1	[0000022]	0	0	0	0	0	2	2
22	[0110101]	0	1	1	0	1	0	1								
23	[1011010]	1	0	1	1	0	1	0								
24	[1011001]	1	0	1	1	0	0	1								
25	[1001101]	1	0	0	1	1	0	1								
26	[0101101]	0	1	0	1	1	0	1								
27	[1010110]	1	0	1	0	1	1	0								
28	[1010011]	1	0	1	0	0	1	1								
29	[1001011]	1	0	0	1	0	1	1								
30	[0101011]	0	1	0	1	0	1	1								
31	[1010101]	1	0	1	0	1	0	1								
32	[1111000]	1	1	1	1	0	0	0								
33	[0111100]	0	1	1	1	1	0	0								
34	[0011110]	0	0	1	1	1	1	0								
35	[0001111]	0	0	0	1	1	1	1								

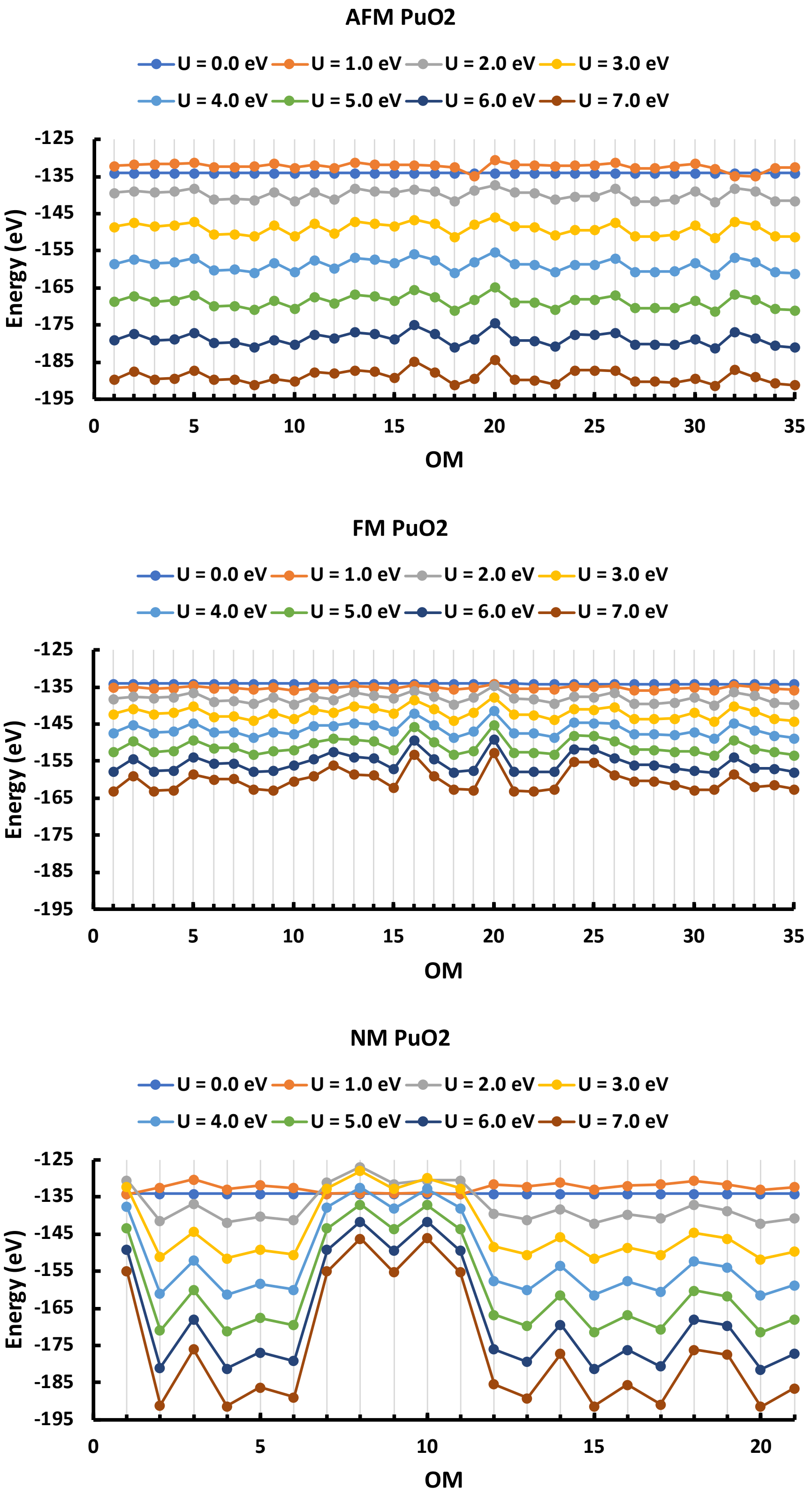

Figure S1: energies of all solutions of AFM, FM and NM PuO_2 as a function of *U* from 0.0 eV to 7.0 eV. OMs are listed in Table S2.

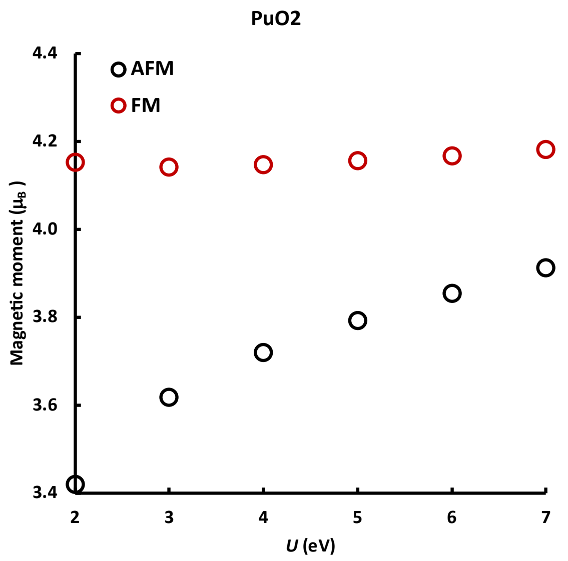

Figure S2: Magnetic moment of Pu in AFM and FM PuO_2.

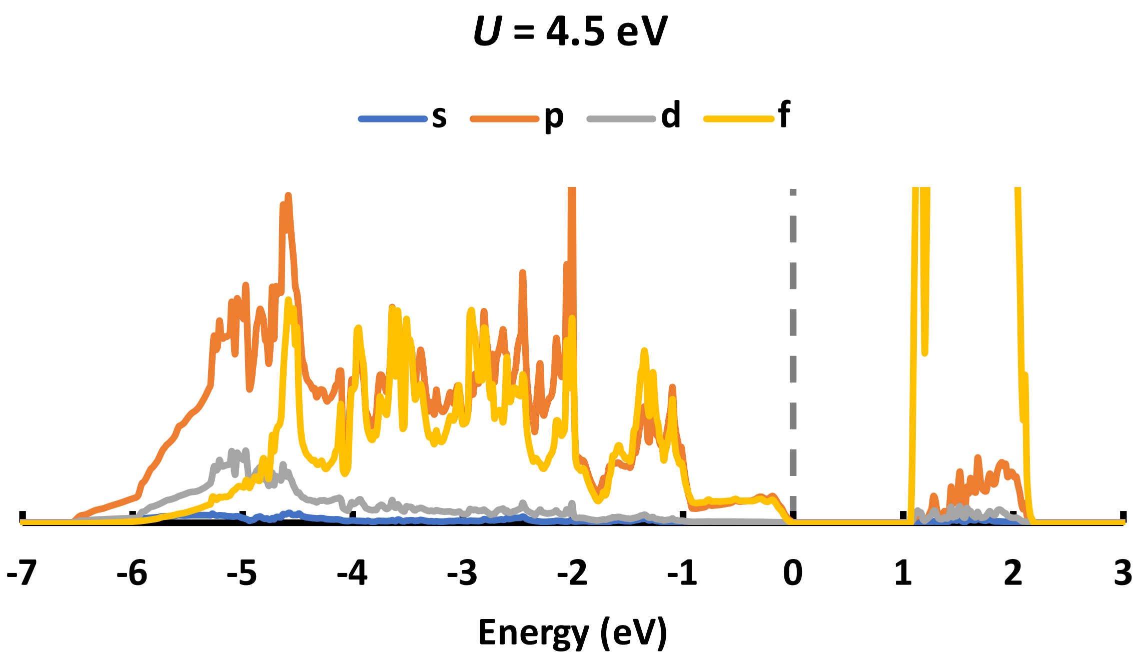

Figure S3: DOS of AFM PuO_2 calculated with PBESol + *U* (4.5 eV) + OMC.

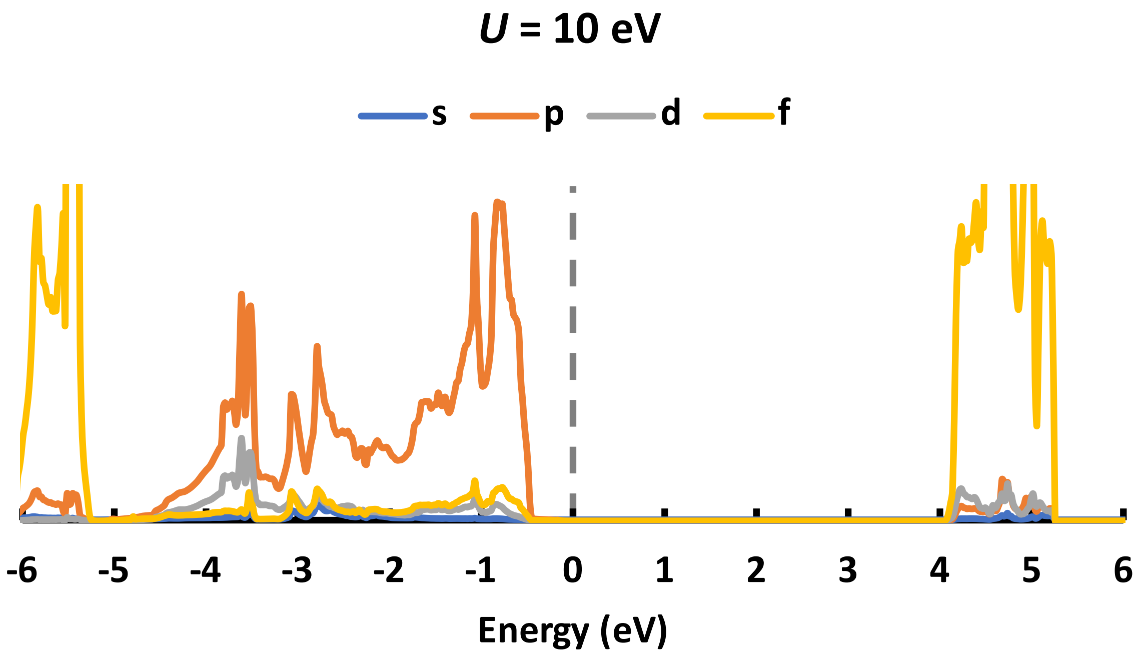

Figure S4: DOS of NM PuO2 calculated with PBESol + *U* (10.0 eV) + OMC.

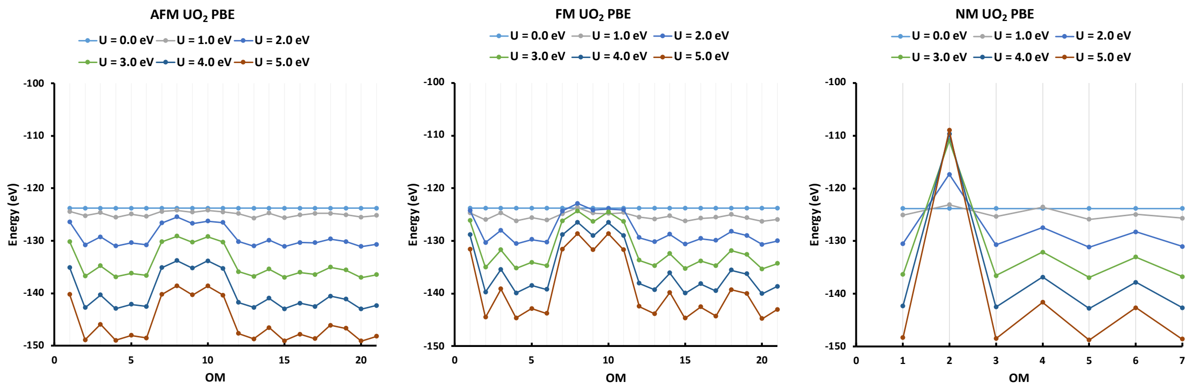

Figure S5: energies of all solutions of AFM, FM and NM UO_2 as a function of U from 0.0 eV to 7.0 eV calculated with the PBE functional. OMs are listed in Table S1.

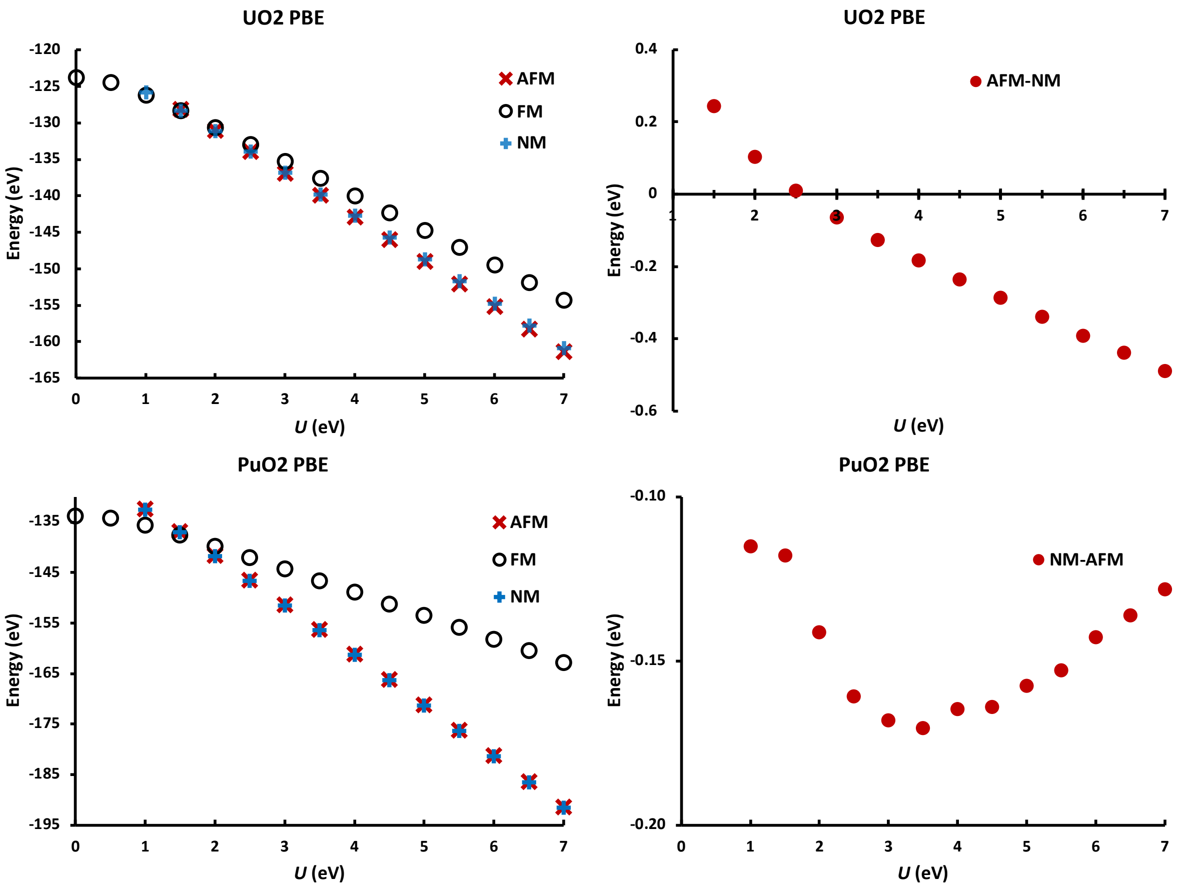

Figure S6: energies of AFM, FM, and NM UO_2 (upper) and PuO_2 (lower) against U value, and the energy difference between the AFM and NM states, all calculated with the PBE functional.

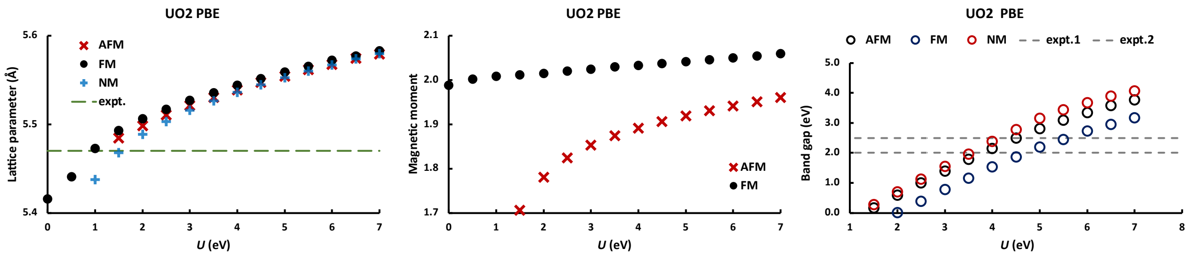

Figure S7: lattice parameter (left), magnetic moment (middle), and band gap (right) of UO_2 against U, calculated using the PBE functional, as well as experimental data from references 32-34 and 42-43 in the main text.

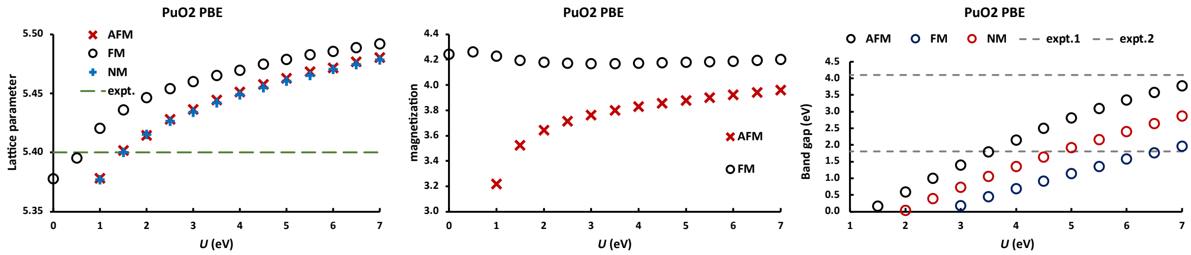

Figure S8: lattice parameter (left), magnetic moment (middle), and band gap (right) of PuO_2 against U, calculated with the PBE functional, as well as experimental data from references 34-48 in the main text.

Table S3: bulk properties of AFM UO_2, NM PuO_2 and AFM PuO_2 from experiments (experimental values for UO_2 from references 32-34 and 42-43 in the main text, experimental values for PuO_2 from references 34-48 in the main text). Data obtained with PBE + U +OMC (U = 4.0 eV for AFM UO_2 and AFM PuO_2, 4.5 eV for NM PuO_2).

	AFM UO_2		NM PuO_2		AFM PuO_2
	Expt.	Cal.	Expt.	Cal.	Cal.
Lattice parameter (Å)	5.470 -5.473	5.54	5.393-5.398	5.45	5.45
Magnetic moment (μ_B)	1.74	1.89	0	0	3.83
Band gap (eV)	2.0 – 2.5	2.15	1.8 – 4.1	1.64	2.15
